# The Identification of Candidate Biomarkers and Pathways in Atherosclerosis by Integrated Bioinformatics Analysis

**DOI:** 10.1155/2021/6276480

**Published:** 2021-11-10

**Authors:** Youwei Lu, Xi Zhang, Wei Hu, Qianhong Yang

**Affiliations:** ^1^Department of Geriatrics, Minhang Hospital, Fudan University, 170 Xinsong Road, Shanghai 201199, China; ^2^Department of Cardiology, Minhang Hospital, Fudan University, 170 Xinsong Road, Shanghai, China 201199

## Abstract

**Background:**

Atherosclerosis (AS) is a type of yellow substance containing cholesterol in the intima of large and middle arteries, which is mostly caused by fat metabolism disorders and neurovascular dysfunction.

**Materials and Methods:**

The GSE100927 data got analyzed to find out the differentially expressed genes (DEGs) using the limma package in R software. Gene Ontology (GO) and the Kyoto Encyclopedia of Genes and Genomes (KEGG) analyses of the DEGs were assessed by the Database for Annotation, Visualization, and Integrated Discovery (DAVID). The Search Tool for the Retrieval of Interacting Genes (STRING) visualized the Protein-Protein Interaction (PPI) network of the aggregated DEGs. GSEA software was used to verify the biological process.

**Result:**

We screened 1574 DEGs from 69 groups of atherosclerotic carotid artery and 35 groups of control carotid artery, including 1033 upregulated DEGs and 541 downregulated DEGs. DEGs of AS were chiefly related to immune response, Epstein-Barr virus infection, vascular smooth muscle contraction, and cGMP-PKG signaling pathway. Through PPI networks, we found that the hub genes of AS were PTAFR, VAMP8, RNF19A, VPRBP, RNF217, KLHL42, NEDD4, SH3RF1, UBE2N, PJA2, RNF115, ITCH, SKP1, FBXW4, and UBE2H. GSEA analysis showed that GSE100927 was concentrated in RIPK1-mediated regulated necrosis, FC epsilon receptor fceri signaling, Fceri-mediated NF KB activation, TBC rabgaps, TRAF6-mediated induction of TAK1 complex within TLR4 complex, and RAB regulation of trafficking.

**Conclusion:**

Our analysis reveals that immune response, Epstein-Barr virus infection, and so on were major signatures of AS. PTAFR, VAMP8, VPRBP, RNF217, KLHL42, and NEDD4 might facilitate the AS tumorigenesis, which could be new biomarkers for diagnosis and therapy of AS.

## 1. Background

Atherosclerosis (AS) is a slow progressive disease that occurs in the coronary arteries, carotid arteries, cerebral arteries, renal arteries, and other large and middle arteries [1]. It is also frequently seen in the cardiovascular and cerebrovascular systems [2]. Approximately 20 million people die from diseases caused by AS each year, and the onset age of AS is becoming younger [3]. The AS symptoms differ from the vascular diseases and the ischemia degree of the involved organs [4]. The etiology of AS is very complex, involving hyperlipidemia, hypertension, smoking, diabetes, obesity, immune damage, and genetic factors [5]. In the early stage of onset, patients generally take drugs and appropriate exercise to control the progression of the disease; those who are severely ill require surgery [6]. Due to the complex etiology of the disease and multiple complications, it is necessary to determine the biomarkers of AS for improving the treatment of patients and reducing the risk of the disease.

The high-throughput gene microarray analysis method has been applied to disease research by more and more researchers, making it possible to analyze the transcriptome and genome of species in a detailed and complete picture [7–10]. Although microarray technology can simultaneously detect thousands of quantitatively expressed gene transcripts from cells or tissues, it lacks reliability and independent statistical analysis [11]. In studying the molecular mechanism of the disease, we need to integrate the best technical means to identify potential diagnostic and therapeutic targets, which will bring great benefits to the diagnosis and therapy of AS.

In this study, we obtained the GSE100927 data which contained 69 groups of the atherosclerotic carotid artery and 35 groups of control carotid artery. Through the GEO2R analysis of these samples, it was concluded that differentially expressed genes (DEGs) were classified into upregulated and downregulated genes. Then, the Database for Annotation, Visualization, and Integrated Discovery (DAVID) was used to enrich the pathways of these DEGs in the Gene Ontology (GO) and Kyoto Encyclopedia of Genes and Genomes (KEGG). Subsequently, based on the online Search Tool for the Retrieval of Interacting Genes (STRING) and Cytoscape, the Protein-Protein Interaction (PPI) network of upregulated DEGs and downregulated DEGs was built and the hub genes were analyzed. Finally, in order to further verify the biological functions of genes in AS patients, the pathways of these genes in REACTOME were enriched and analyzed by Gene Set Enrichment Analysis (GSEA). The above research results may provide effective biomarkers for AS patients and improve AS therapies.

## 2. Materials and Methods

### 2.1. Microarray Data

We downloaded GSE100927 data from the Gene Expression Omnibus (GEO, http://www.ncbi.nlm.nih.gov/geo) database and saved it in TXT format. The GSE100927 gene expression profile contained a total of 104 data samples, which were 69 groups of the atherosclerotic carotid artery and 35 groups of control carotid artery, which was used as a basis for subsequent research.

### 2.2. Identification of DEGs

In this study, the GEOquery and limma R packages in the Bioconductor project can be used to analyze and process the raw data [12]. We set *P* < 1*e* − 12 and FC > 1 as the selection criteria for upregulated DEGs and *P* < 1*e* − 12 and FC < 1 for downregulated DEGs. The subsequent results were displayed using the volcano map made by ImageGP.

### 2.3. Enrichment Analysis of GO and KEGG Pathways of DEGs

GO is a database established by the Gene Ontology Consortium that applies to various species and provides a limited description of gene or protein functions. It is categorized into three parts: Cellular Component (CC), Molecular Function (MF), and Biological Process (BP) [13]. KEGG is a pathway-related database that integrates genomic, chemical, and system function information [14]. In this study, we used the DAVID (https://david.ncifcrf.gov) database to delve into the BP of DEGs in GO and the pathway in KEGG.

### 2.4. Construction of the PPI Network and MCODE Plugin Analysis

The STRING (https://string-db.org/) database could seek for interactions between known proteins and predicted protein interactions [15]. In order to explore the relationship between DEGs, we separately evaluated the upregulated and downregulated DEGs based on the STRING database. After that, based on Cytoscape (https://cytoscape.org/), a graphical display network software for analysis and editing, we built PPI networks for DEGs. Finally, we analyzed the MCODE plugin to determine the hub genes.

### 2.5. GSEA

REACTOME (https://reactome.org/) is a signal pathway database similar to KEGG, which provides users with knowledge visualization, interpretation, and analysis of bioinformatics [16]. GSEA is a gene-by-gene comparison technique for genome-wide expression profile chip data [17]. To further explore the potential functions of genes in AS patients, we analyzed the enrichment of genes in the REACTOME pathway.

## 3. Results

### 3.1. Identification of DEGs

**Figure 1:**
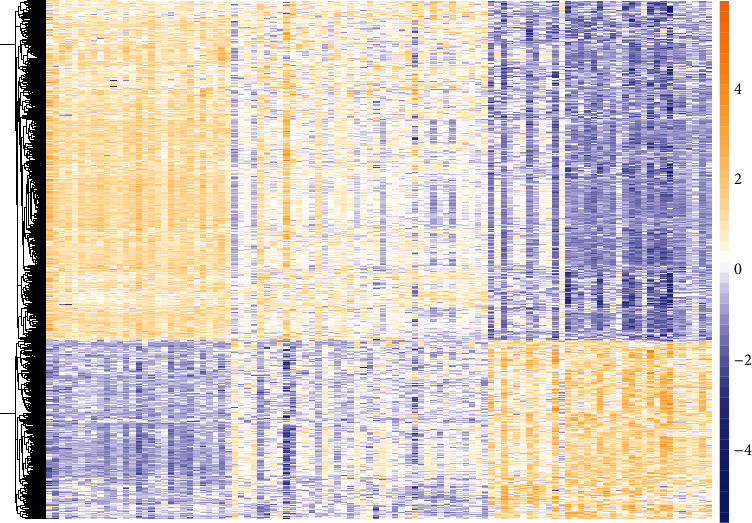
Heat map of GSE100927 gene expression profile. The color from yellow to blue indicates the expression of DEGs from high to low.

The GSE100927 gene expression profile we downloaded contained 69 groups of atherosclerotic carotid artery and 35 groups of control carotid artery. According to the adjusted *P* value and the filtering conditions of FC, we identified 1033 upregulated DEGs and 541 downregulated DEGs. The top 10 most significant upregulated DEGs were IBSP, MMP9, ACP5, CHI3L1, CCL3L3, IGLL5, DHRS9, CCL3, SPP1, and SLAMF7, respectively. The top 10 most significant downregulated DEGs were PLA2G2A, MYOC, PI16, SCARA5, VIT, APOD, HSPB7, WNT11, XLOC_013983, and AOX1, respectively.  Figure 1 shows the cluster distribution of 1574 DEGs in 104 samples.

### 3.2. GO and KEGG Enrichment Analyses of Upregulated DEGs

**Figure 2:**
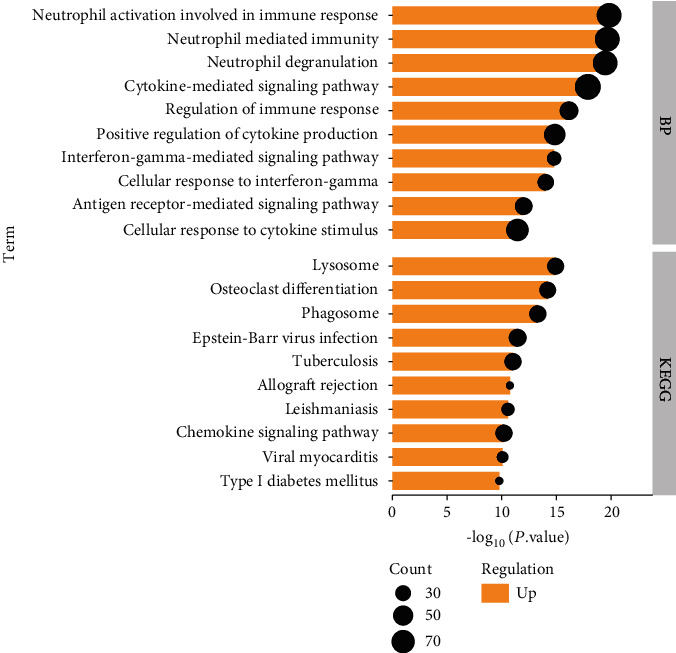
BP and KEGG enrichment analyses of upregulated DEGs. The abscissa represents the *P* value, and the ordinate represents the BP terms and KEGG channels.

Through the enrichment analyses of GO and KEGG on the upregulated DEGs, we could conclude from the results in Figure 2 that these DEGs were enriched in BP in neutrophil activation involved in immune response, neutrophil-mediated immunity, neutrophil degranulation, cytokine-mediated signaling pathway, regulation of immune response, positive regulation of cytokine production, cellular response to interferon-gamma, cellular response to cytokine stimulus, and antigen receptor-mediated and interferon-gamma-mediated signaling pathways. In KEGG, they are enriched in lysosome, osteoclast differentiation, phagosome, Epstein-Barr virus infection, tuberculosis, allograft rejection, leishmaniasis, chemokine signaling pathway, viral myocarditis, and type I diabetes mellitus.

### 3.3. GO and KEGG Enrichment Analyses of Downregulated DEGs

**Figure 3:**
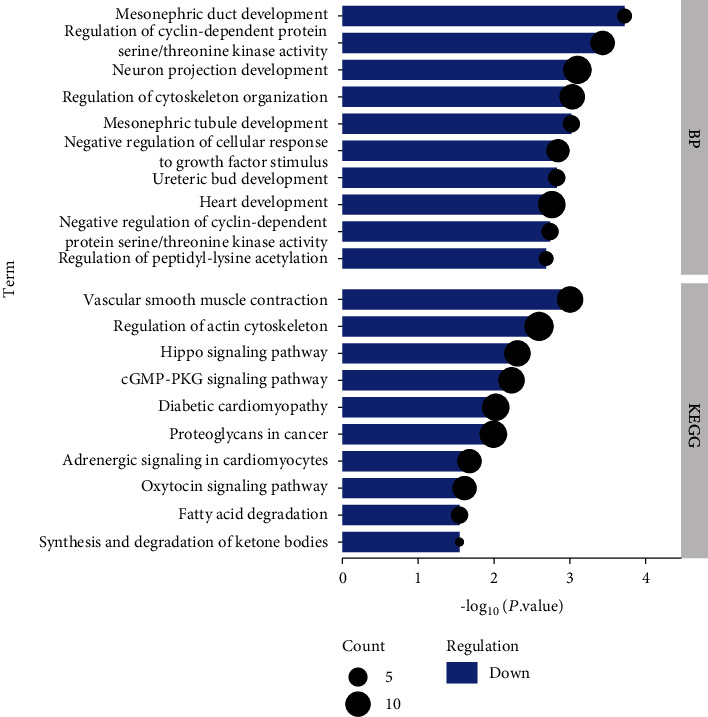
BP and KEGG enrichment analyses of downregulated DEGs. The abscissa represents the *P* value, and the ordinate represents the BP terms and KEGG channels.

In BP, downregulated DEGs gathered in mesonephric duct development, neuron projection development, regulation of cytoskeleton organization, mesonephric tubule development, ureteric bud development, heart development, negative regulation of cyclin-dependent protein serine/threonine kinase activity and cellular response to growth factor stimulus, regulation of peptidyl-lysine acetylation, and cyclin-dependent protein serine/threonine kinase activity (Figure 3). It could be seen from the results in Figure 3 that the downregulated DEGs enriched ten pathways, namely, vascular smooth muscle contraction, regulation of actin cytoskeleton, diabetic cardiomyopathy, proteoglycans in cancer, adrenergic signaling in cardiomyocytes, fatty acid degradation, synthesis and degradation of ketone bodies, Hippo, cGMP-PKG, and oxytocin signaling pathways.

### 3.4. PPI Network of Upregulated DEGs and Identification of Hub Genes

**Figure 4:**
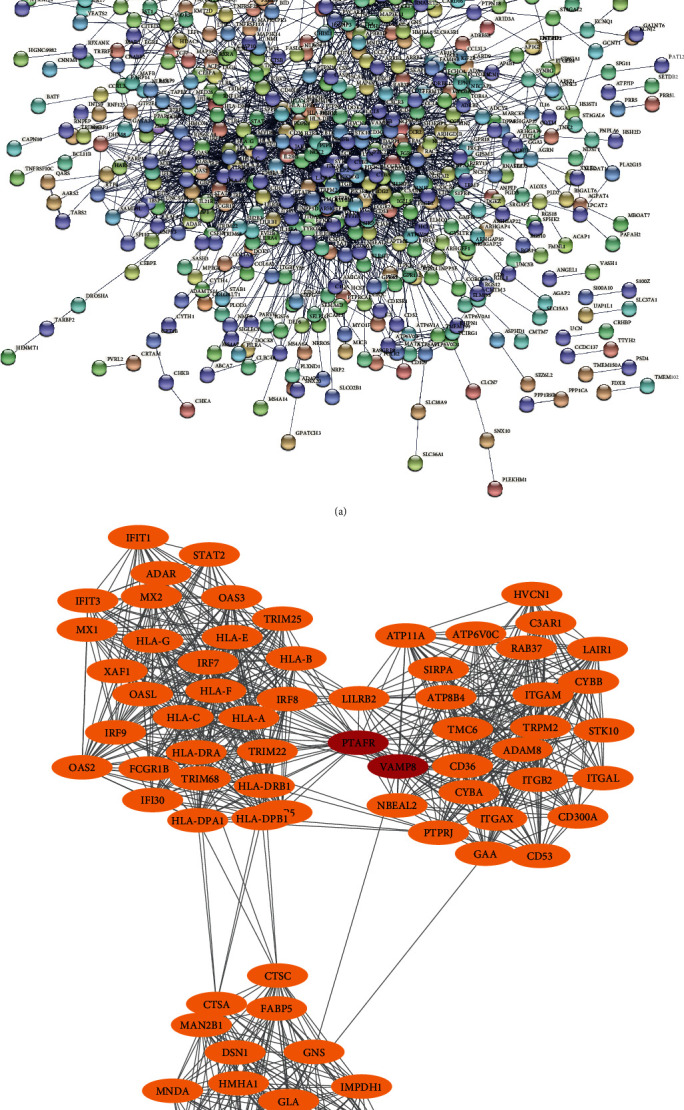
Analysis of PPI network and modules for upregulation of DEGs. (a) PPI network constructed by Cytoscape. (b) Important modules obtained by MCODE plugin. The upregulated hub genes are RTAFR and VAMP8.

Figure 4(a) is the PPI network constructed by upregulated DEGs. The network consisted of 608 nodes and 3121 edges. Due to too many genes involved, in order to screen out hub genes more accurately, we selected genes with high degree scores for MCODE analysis. According to Figure 4(b), it could be seen that the graph has 76 nodes and 876 edges. The first two genes with the highest score value were identified as the upregulated hub genes, which were PTAFR (degree = 41) and VAMP8 (degree = 33).

### 3.5. PPI Network of Downregulated DEGs and Identification of Hub Genes

**Figure 5:**
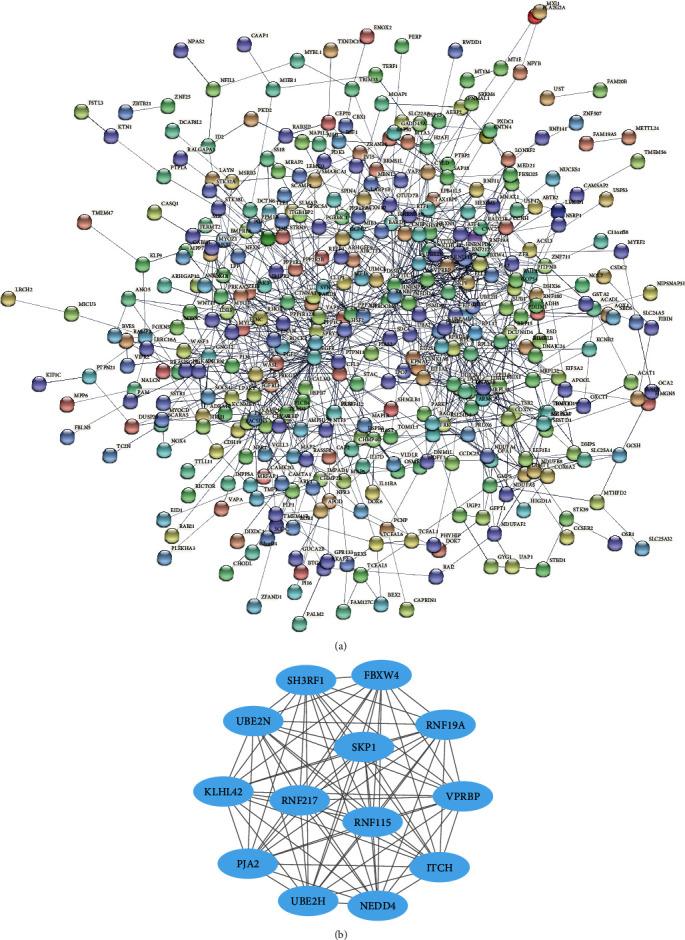
Analysis of PPI network and modules for downregulation of DEGs. (a) PPI network constructed by Cytoscape. (b) Important modules obtained by MCODE plugin. The downregulated hub genes are RNF19A, VPRBP, RNF217, KLHL42, NEDD4, SH3RF1, UBE2N, PJA2, RNF115, ITCH, SKP1, FBXW4, and UBE2H.

The PPI network of downregulated DEGs was composed of 387 nodes and 10001 edges (Figure 5(a)). In order to screen out the hub genes that are downregulated DEGs, we selected 13 genes to form an interactive network with a total of 13 nodes and 78 edges (Figure 5(b)). On the basis of these 13 genes, we found that the degrees of RNF19A, VPRBP, RNF217, KLHL42, NEDD4, SH3RF1, UBE2N, PJA2, RNF115, ITCH, SKP1, FBXW4, and UBE2H were all 12. Therefore, these 13 genes could be regarded as downregulated hub genes.

### 3.6. GSEA

**Figure 6:**
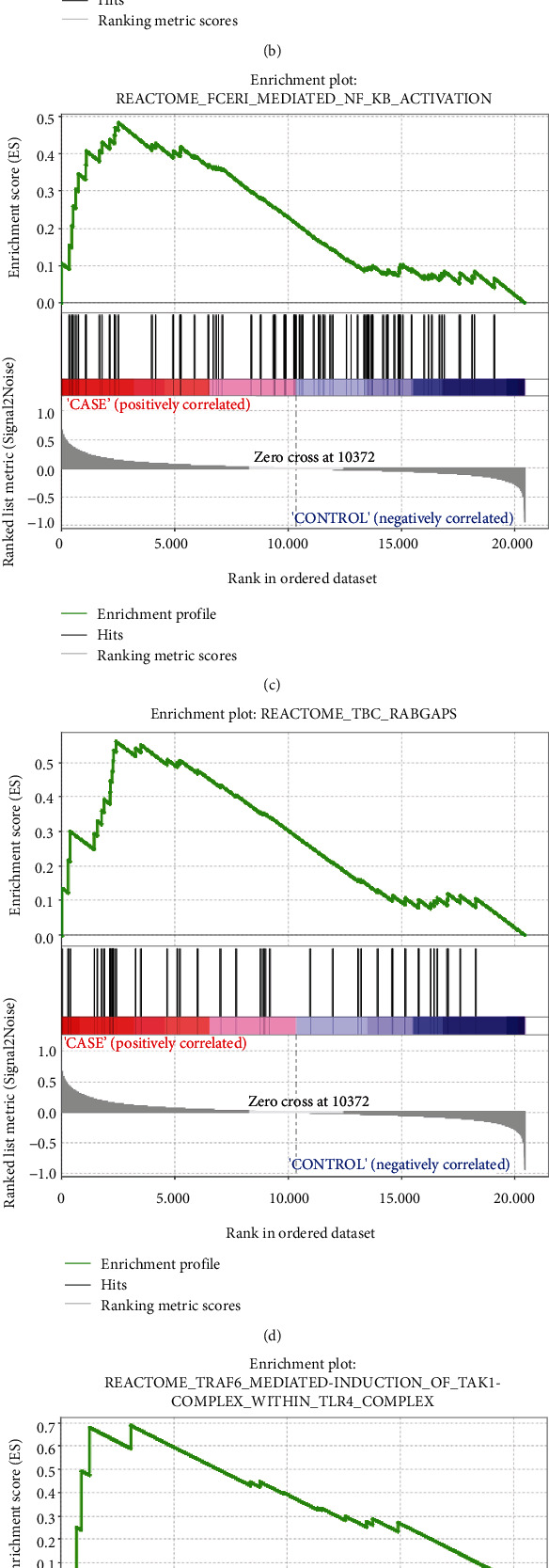
Enrichment analysis of the REACTOME pathway based on GSEA: (a) RIPK1-mediated regulated necrosis; (b) FC epsilon receptor fceri signaling; (c) Fceri-mediated NF KB activation; (d) TBC rabgaps; (e) TRAF6-mediated induction of TAK1 complex within TLR4 complex; (f) RAB regulation of trafficking.

In order to have a deeper understanding of the gene function in AS patients, we conducted an enrichment analysis of the REACTOME pathway through GSEA. According to Figures 6(a)–6(f), the genes in AS patients were enriched in 6 pathways, namely, RIPK1-mediated regulated necrosis, FC epsilon receptor fceri signaling, Fceri-mediated NF KB activation, TBC rabgaps, TRAF6-mediated induction of TAK1 complex within TLR4 complex, and RAB regulation of trafficking.

## 4. Discussion

AS refers to the yellowish atherosclerotic lipids accumulating in the artery intima; it brings multiple complications like coronary heart disease [18]. The characteristic is that the affected arteries start from the intima. If the patient's condition is mild, it generally does not affect the quality of life and life expectancy [19]. However, the prognosis of AS must be closely related to the complications of the heart, brain, and kidneys. Severe AS can endanger major organs, such as angina pectoris, acute myocardial infarction, heart failure, renal perfusion blood flow reduction and kidney necrosis, cerebral infarction, peripheral AS, and aortic aneurysm [20]. Due to the various difficulties and uncertainties in the diagnosis and treatment of AS, the prognosis of AS is often poor [21]. Therefore, it is urgent to find key genes and signal pathways related to improve the AS diagnosis efficiency and cure rate.

Through analysis, we discovered that DEGs in AS were mainly involved in immune response, Epstein-Barr virus infection, vascular smooth muscle contraction, and cGMP-PKG signaling pathway. Samson et al. proposed that AS was featured with chronic inflammation and changes in the immune response [22]. The modification of low-density lipoprotein would lead to variation in its function and activate the innate immune system and adaptation [23]. Previous studies have shown that Epstein-Barr virus (EBV) is a ubiquitous oncogenic virus, which causes about 2% of cancers to occur by regulating the activities of a variety of host cells [24]. Early-life EBV infection can result in infectious mononucleosis, a self-limiting lymphoproliferative illness [25]. Huang et al. believed that EBV infection was related to the development of human malignancies [26]. Xiong et al. found that the senescence and apoptosis of vascular smooth muscle cells were involved in the vulnerability of atherosclerotic plaques. Stable plaques were characterized by low vascular smooth muscle cell content and low extracellular matrix content. Unstable plaque rupture with coronary thrombotic occlusion was a fatal complication of AS, which could result in acute coronary syndromes [27]. Studies by Kovács et al. have shown that the cGMP-PKG signaling pathway mediated many processes, such as regulating the relaxation and contraction of vascular smooth muscle cells, antiheart hypertrophy, anti-AS, and antivascular injury/restenosis [28]. The importance of CGMP-PKG pathway regulation is confirmed by more and more evidence.

In our study, we aimed to identify biomarkers of AS and uncover their biological functions through constructing PPI networks. Through the Cytoscape software plugin, we have identified that the hub genes related to AS were mainly PTAFR, VAMP8, VPRBP, RNF217, KLHL42, and NEDD4. Many studies have shown that PTAFR (platelet-activating factor receptor) promotes tumorigenesis, angiogenesis, and metastasis [29]. In addition to participating in the production of cytokines and chemokines, PTAFR plays an active role in the progression of atherosclerotic plaques. Chen et al. pointed out that VAMP8 (vesicle-associated membrane protein 8) was a trap and had been found in a variety of important cellular activities [30]. VAMP8 is significantly overexpressed in human glioma specimens and can be used as a new indicator of glioma prognosis and treatment [31].

The GSEA results based on all gene expression information revealed that gene set GSE100927 was significantly enriched in 6 pathways, namely, RIPK1-mediated regulated necrosis, FC epsilon receptor fceri signaling, Fceri-mediated NF KB activation, TBC rabgaps, TRAF6-mediated induction of TAK1 complex within TLR4 complex, and RAB regulation of trafficking. Meng et al. pointed out that RIPK1 was an important mediator of cell death and inflammatory response downstream of TNFR1 [32]. Under the stimulation of TNF-*α*, TNFR1 was a powerful proinflammatory cytokine that participates in the occurrence of various inflammations and degenerative diseases [33]. RIPK1-DD-mediated dimerization was the key to promote the activation of RIPK1 during the process of tumor necrosis factor-*α*-stimulated cell transition from complex I to complex II [34]. In addition, the potential crucial genes need further validation by RT-qPCR in clinical samples. Finally, the mechanisms in which these genes play are not completely clear. More evidence is required to find out the biological foundation.

## 5. Conclusion

In summary, our research identified 1574 DEGs, including 1033 upregulated DEGs and 541 downregulated DEGs. Functional and pathway enrichment analyses exhibited that DEGs of AS were concentrated in immune response, Epstein-Barr virus infection, vascular smooth muscle contraction, and cGMP-PKG signaling pathway. The hub genes of AS were PTAFR, VAMP8, RNF19A, VPRBP, RNF217, KLHL42, NEDD4, SH3RF1, UBE2N, PJA2, RNF115, ITCH, SKP1, FBXW4, and UBE2H. GSEA analysis revealed RIPK1-mediated regulated necrosis, FC epsilon receptor fceri signaling, Fceri-mediated NF KB activation, TBC rabgaps, TRAF6-mediated induction of TAK1 complex within TLR4 complex, and RAB regulation of trafficking. These hub genes and signaling pathways may be related to the occurrence and development of AS and can be used to determine biomarkers of AS and explore the treatment of AS.

## Data Availability

The datasets used and/or analyzed during the current study are available from the corresponding author on reasonable request.
